# Insight into loading, release, and anticancer activities of the methanol hybridized glauconite nano-sheets as a potential carrier of cisplatin: equilibrium and release kinetics

**DOI:** 10.3389/fchem.2025.1523664

**Published:** 2025-02-27

**Authors:** Haifa E. Alfassam, Nourhan Nasser, Sarah I. Othman, Hanan M. Alharbi, Noof A. Alenazi, Hassan A. Rudyani, Ahmed A. Allam, Wail Al Zoubi, Mostafa R. Abukhadra

**Affiliations:** ^1^ Department of Biology, College of Science, Princess Nourah bint Abdulrahman University, Riyadh, Saudi Arabia; ^2^ Materials Technologies and their Applications Lab, Geology Department, Faculty of Science, Beni-Suef University, Beni Suef, Egypt; ^3^ Department of Chemistry, College of Science and Humanities in Al-Kharj, Prince Sattam bin Abdulaziz University, Al-Kharj, Saudi Arabia; ^4^ Department of Biology, College of Science, Imam Mohammad Ibn Saud Islamic University, Riyadh, Saudi Arabia; ^5^ Materials Electrochemistry Laboratory, School of Materials Science and Engineering, Yeungnam University, Gyeongsan, Republic of Korea; ^6^ Applied Science Research Center, Applied Science Private University, Amman, Jordan

**Keywords:** glauconite, exfoliation, methoxy, cisplatin, loading, release, cytotoxicity

## Abstract

Advanced silicate nano-sheets as exfoliated and separated layers were developed from natural glauconite and hybridized with methanol, producing a methoxy exfoliated structure (Mth/EXGL). The structure was assessed as an enhanced carrier of the cisplatin drug (CSPN) with significant loading, release, and cytotoxicity properties. The methoxy form of exfoliated glauconite showed better loading properties (327.7 mg/g) than the exfoliated sample (202.4 mg/g) as well as the raw sample (119.3 mg/g). This enhancement was assigned to the incorporated active loading centers after the methanol hybridization step, which is in agreement with the steric studies and determined active site density (Nm = 45.5 mg/g (Mth/EXGL), 38.4 mg/g (EXGL), and 26.3 mg/g (glauconite). Moreover, each site across the interface of Mth/EXGL has the capacity to be loaded with 8 CSPN molecules, donating multi-molecular mechanisms and their loading in vertical orientation. The CSPN loading energy value (<8 kJ/mol) into Mth/EXGL reflected the dominant impact of the physical mechanisms, including electrostatic attractions and hydrogen bonding. The recognized release profile demonstrates continuous and controlled behavior that can extend up to 110 h at pH 7.4 and 170 h at pH 5.5. This releasing behavior is regulated by two main processes (diffusion and erosion) based on the release kinetic findings. Also, Mth/EXGL as a carrier of CSPN induces its cytotoxic effect on human cervical epithelial tumors (HeLa) (0.65% cell viability) as compared to the free form of CSPN (6.6% cell viability). The Mth/EXGL is recommended as a delivery system for CSPN considering its determined loading, release, and cytotoxicity properties.

## 1 Introduction

Noncommunicable diseases (NCDs), which arise from a combination of genetic, physiological, environmental, and behavioral factors, currently account for 71% of all global deaths. Among these, cancer ranks as the second leading cause of mortality associated with NCDs (World Health Organization, 2020). Projections indicate that by 2040, approximately 16.4 million individuals are expected to die from cancer, representing a 71.1% increase compared to the estimates from 2018 (Ferlay et al., 2019). Chemotherapy remains the most commonly employed treatment strategy for cancer. However, despite significant advancements in this field, chemotherapy has several limitations, including low therapeutic efficacy, poor bioavailability, and non-specific drug distribution, which significantly reduces its effectiveness against tumors (Nandi et al., 2020; Raj et al., 2016).

Patients with carcinoma frequently utilize a variety of medications that inhibit the uncontrollable proliferation of cancerous cells, which improves the quality of their health and extends their life duration (Ferlay et al., 2019; Raj et al., 2016; Lee et al., 2019; Li et al., 2021). Regrettably, the majority of chemotherapeutic medications currently in use negatively impact healthy cells and have the potential to cause serious side effects, particularly when administered in excess (Cutrim et al., 2021; Chen et al., 2024). Producing affordable medications with physiologically active chemical frameworks and safe drug delivery structures is an important priority for academics and interested health organizations. These therapies have been developed to be administered immediately to the position of the carcinomas, resulting in the death of leftover cancer cells and inhibiting their proliferation to nearby tissues. It is essential to verify that these treatments do not have any significant detrimental impacts on the general health of the organisms (Solomevich et al., 2020).

Cisplatin (CSPN), or cis-diamminedichloroplatinum (II), is a commonly employed and economically efficient cancer inhibitor that has a strong therapeutic effect. It is administered as a kind of chemotherapeutic medication that is widely used during the treatment of a variety of tumors, including testicular, ovarian, head, neck, pulmonary, and bladder tumors (Dasari and Bernard Tchounwou, 2014; Zoń and Bednarek, 2023). Cisplatin is classified as an alkylating chemotherapy medication that exerts its effects on cells at all stages of their biological cycle, independent of their specific phase. Its medicinal properties are attributable to the formation of crosslinks inside the purine strand of DNA in tumor cells, which inhibit DNA formation and eventually destroy cells (Li and Lin, 2023; Kaidar-Person et al., 2018). Unfortunately, the consumption of cisplatin throughout the chemotherapy course has been correlated with a variety of prospective adverse side effects that commonly occur following the injection of the cytostatic drugs, including elevated toxicity and nephrotoxicity (Hamaya et al., 2023; Oun et al., 2018). In the last few years, several effective methods of administration have been successfully developed to reduce these negative impacts. For this particular objective, a number of drivers were evaluated, comprising lipids, metallic nanoparticles, micelles, polymers, and cryogels (Solomevich et al., 2020).

Most of the vehicles or carriers that are employed significantly boost both the penetration and retention properties of chemotherapy drugs. Several investigates have thoroughly characterized clay minerals, including kaolinite, sepiolite, vermiculite, montmorillonite, and halloysite, as very effective transporters of frequently used chemotherapeutic agents (Swain et al., 2024a). Clay minerals typically consist of stratified aluminosilicate sheets which have considerable ion exchange functions, lack of toxic effects, biologically compatible, chemically stable, have significant ability to adsorb soluble chemical and ions, are inexpensive, thermally stable, and have adaptable chemical compositions (Song et al., 2024; Almahri, 2022a; Swain et al., 2024b; Ali et al., 2024). Additional studies were conducted to enhance the exterior chemistry, biology, and physicochemical properties of widely employed clay minerals by applying different modifying approaches. These procedures encompass alkaline chemical modifications, thermal treatment, acid activation, pillaring with metal ions, blending with metal oxides, exfoliating, integrating with polymers, scrolling, and organic hybridization using chemicals including CTAB and starch (Almahri, 2022b; Meng et al., 2020; Gong et al., 2022).

Recent studies have highlighted the enhanced physicochemical and biological characteristics of methoxy-modified nanostructures as a form of modified clay (Ashiq et al., 2021; Tan et al., 2018). Through the implementation of environmentally safe grafting methods, methanol molecules can be inserted between the layers of clay structures that contain various hydroxyl groups, specifically across the inner surface (Tan et al., 2018; Tchoumene et al., 2022; Li et al., 2017). Previous research was primarily focused on analyzing the technical properties of methoxy-modified varieties of kaolinite in combination with bentonite. Nevertheless, there has been a deficiency of studies examining the distinguishing characteristics of the methoxy versions corresponding to other species of clay minerals, such as glauconite (Ashiq et al., 2021; Borah et al., 2023). Glauconite is a naturally occurring clay mineral, recognized by its chemical composition as a potassium-ferric phyllosilicate (K, Na) (Fe^3+^Fe^2+^, Al, Mg)_2_(Si,Al)_4_O_10_(OH)_2_). Glauconite consists of successive layers of illite and smectite, each containing an alumina di-octahedral unit embedded between two silica tetrahedron units. Additionally, these consecutive layers contain potassium cations throughout the interlayer spaces. Glauconite, as a mineral, has abundant natural availability, low costs, a chemical structure enriched with metals, an attractive geometry, a large surface area, practicable catalytic properties, and efficient ion exchange qualities (Singla et al., 2020a; Shmandiy et al., 2020). As a result, it possesses an extensive amount of replaceable hydroxyl groups, which makes it less difficult to accommodate the alcoholic compounds within its multilayered structure. The aforementioned characteristic allows methanol to develop bonds with glauconite active groups without the need to conduct extra pre-treatment processes (Ashiq et al., 2021). Therefore, it was predicted that the embedding of methanol molecules between the glauconitic layers might result in a hybrid framework containing numerous functions and enhanced physicochemical properties (Borah et al., 2023).

Moreover, the study of clay’s morphological transformations has improved significantly in the past few years, particularly in the field of separating or exfoliating the structurally stratified units into separate nano-silicate sheets (Alqahtani et al., 2023a; Abdo et al., 2022). The successful implementation of this modification technique resulted in distinctive and innovative nanomaterials developed from clay. These structures possess distinctive advantages, including potent biological activities, high adsorption potential, strong oxidation properties, a reactive external interface, efficient dispersion activities, and a large surface area (Abukhadra et al., 2024a; Tang et al., 2023; Abukhadra et al., 2024b). Therefore, the production of methoxy derivatives of the separated and stripped glauconite nanosheets will probably yield much enhanced materials with promising loading and release behaviors as drug delivery structures. As a result, the presented investigation included the first investigation of methoxy exfoliated glauconite (Mth/EXG) as enhanced drug delivery system for cisplatin drug (CSPL) based on synergetic or comparative studies including raw glauocnite and exfoliated glauocnite. This included the detailed loading properties and behaviors, the release profiles and mechanisms, and their anticancer activities against human cervical epithelial tumors (HeLa).

## 2 Experimental work

### 2.1 Materials

The glauconite ore has been obtained through the El-Gedida site in the El-Bahariya Oasis, located in the Western Desert of Egypt. The sample that was evaluated had a chemical composition comprised of SiO_2_ (52.2%), Al_2_O_3_ (6.12%), Fe_2_O_3_ (23.14%), SO_3_ (0.17%), K_2_O (6.48%), Na_2_O (0.08%), MgO (3.53%), TiO_2_ (0.11%), CaO (0.27%), P_2_O_5_ (0.10%), MnO (0.01%), and a loss on ignition (L.O.I.) of 7.8%. The presented major oxides were measured using Panalytical Axios Advanced XRF device in the Nuclear Material Authority of Egypt. The chemicals implemented throughout the glauconite transformation steps have been purchased from Sigma-Aldrich in Egypt, including cetyltrimethylammonium bromide (CTAB) (above 98%), dimethyl sulfoxide (DMSO) (above 99.5%), sodium hydroxide pellets (97%), and methanol (above 99.9%). The anticancer medicine utilized was a cisplatin drug (cis-Diammineplatinum (II) dichloride; (Pt (NH_3_)_2_Cl_2_) with a purity of ≥98% and delivered by Sigma-Aldrich company (CAS Number: 15,663–27–1).

### 2.2 Synthesis of methoxy exfoliated glauconite (Mth/EXGL)

The EXGL was successfully produced according to the procedures described by Abukhadra et al. (Abukhadra et al., 2024a). The original glauconite, a natural mined mineral, was ground to a fine powder and mixed homogenously within 200 mL of a dilute DMSO mixture consisting of 80% DMSO and 10% water. The entire mixture was then forcefully agitated for a duration of 72 h. This process is vital to destroying the hydrogen bonds between the layers of clay. Afterwards, the resultant product was subjected to five rounds of methanol rinsing, with each round taking approximately 20 min. The step led to the development of methoxy glauconite, with a marked exfoliation effect on the multilayered silicate units. Subsequently, the Mth/EXGL particles were obtained by filtration, followed by several rinsing cycles using distilled water, and finally dried at a temperature of 60°C for 10 h. Then the sample was immersed in methanol for an additional 48 h to ensure the formation of methoxy-modified forms (Mth/EXG).

### 2.3 Characterization instruments

The structural and crystalline properties of the materials have been determined by examining their X-ray diffraction (XRD) features using a PANalytical-Empyrean X-ray diffractometer, considering the detecting angles between 0° and 70°. The Shimadzu FTIR-8400S spectrometer was used to assess changes in the fundamental chemical groups across the entire production process. The spectrometer possesses a measurement range spanning from 400 to 4,000 cm^−1^. The surface geometries and morphologies were analyzed using a Gemini Zeiss Ultra 55 scanning electron microscope. Before imaging, the surfaces of the materials being examined were coated with thin gold coatings. In addition, their interiors were analyzed using HRTEM photographs taken using a transmission electron microscope (JEOL-JEM2100) at an accelerating voltage of 200 kV. The surface area analyzer (Beckman Coulter SA3100) was used to measure the specific surface area.

### 2.4 The loading properties of CSPN

The loading properties of GL, EXGL, and Mth/EXGL were evaluated to determine their potential as drivers for CSPN. This was accomplished by analyzing the effects of different loading conditions. The parameters encompass a pH that varies from 3 to 9, loading durations extending from 1 to 24 h, and drug content that varies from 100 to 600 mg/L. The GL, EXGL, and Mth/EXGL particles had been thoroughly homogenized with the CSPN solutions employing vortex rotator equipment. Whatman filter paper was utilized to extract the GL, EXGL, and Mth/EXGL particles that contained CSPN from the remaining CSPN solutions after completing the loading steps. The remaining content of CSPN inside the filtrates has been measured by a UV-Vis spectrophotometer set to a measurement wavelength of 240 nm. The loading efficiency has been calculated using Equation 1. The loading assessments were performed by means of three distinct tests, and the computations and figures were derived assuming the average values obtained for all three investigations had standard deviations below 5.5%.

$$\text{Loaded drug }\left(\text{mg}/g\right)=\frac{\left(\text{Initial}\;\text{concentration}-\text{Residual}\;\text{concentration}\right)X\;\text{solvent}\;\text{volume}}{\text{Carrier}\;\text{weight}}$$ (1)

### 2.5 The *in vitro* release studies

The tests examined the diffusion properties of CSPN from the GL, EXGL, and Mth/EXGL particles under specific conditions, maintaining the temperature at 37°C ± 1°C. The release responses of CSPN have been examined using an acetate buffering solution having a pH of 4.5 and a phosphate buffering solution having a pH of 7.4. In order to conduct release studies, specific amounts of GL, EXGL, and Mth/EXGL particles containing CSPN (100 mg/g) were separately inserted within 250 mL of the respective buffering solutions. The particles have been mixed inside the buffers employing the DISTEK dissolving equipment for 200 h with a vessel rotating at a speed of 200 rpm. Periodically, 3 mL of each buffered solution was expelled from the containers in order to measure the contents of the liberated CSPN. The quantities of CSPN medications emitted have been determined using a UV-visible spectrophotometer, specifically at a wavelength of 240 nm. To ensure consistent levels of the solutions throughout the *in vitro* release investigations, the collected buffers (3 mL) were reintroduced into the vessels that originally contained the whole volumes of the buffered solutions after each round of measurement. The release assessments were performed three times, and the mean data were applied as the basis of the mathematical computations, with standard deviations below 3.85%. Equation 2 has been used to evaluate the percentage of medication that was released by measuring the CSPN concentrations.

$$\text{Drug release }\left(\%\right)=\frac{\text{The}\;\text{amount}\;\text{of}\;\text{Released}\;\text{drug}\;}{\text{Amount}\;\text{of}\;\text{loaded}\;\text{drug}}\times 100$$ (2)

### 2.6 *In vitro* cytotoxicity

Cell toxicity of free GL, EXGL, and Mth/EXGL, as well as their CSPN-loaded forms, was evaluated using human cervical epithelial malignancies (HeLa) which were obtained from American Type Culture collection (ATCC, MD, United States). The cell cultures were propagated within RPMI media supplemented with L-glutamine (2 mM), fetal bovine serum (10% FBS), and penicillin-streptomycin (1%). To induce the inhibition of cell reproduction, cancer cells were transferred into 96-well plates at a density of about 10,000 cells per well. Each well was filled with 100 μL of the formulated medium. The CSPN, GL, EXGL, and Mth/EXGL structures as free components, together with their CSPN-loaded forms, were blended with the incubation medium to attain cytostatic doses of 0.50 and 1.00 μg/mL. The pH values of the mixture ranged from 6.8 to 7.4. The collected samples were then kept under specific conditions at a constant temperature of 37°C ± 0.2°C before being injected into the cultivated cells. The optical densities of the free and loaded forms were measured at a wavelength of 450 nm across different time frames. The extent of inhibition of cell growth triggered by the materials under investigation has been calculated using the formula Nex/NcX100%, where Nex denotes the median number of cells in the experimental group and Nc reflects the mean number of cells in the control group.

### 2.7 Statistical analysis

The tests have been performed for three repetitions, and the results have been provided in the mean ± standard deviations (SD) (S.E.M.; n = 3). The statistical evaluation’s significance was determined by analyzing the findings of variance (ANOVA) and paired examinations, implementing the value of *p < 0.05.

## 3 Results and discussion

### 3.1 Characterization of the carriers

**FIGURE 1:**
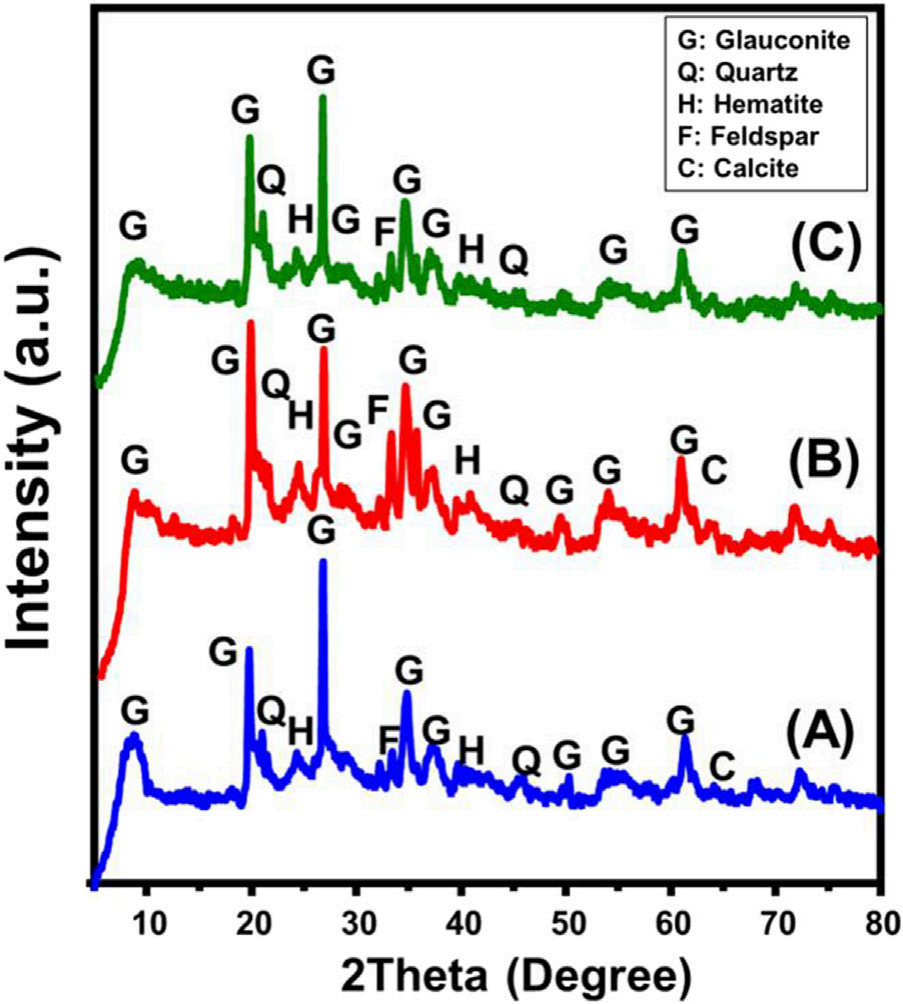
XRD pattern of untreated glauconite **(A)** DMSO modified glauconite **(B)**, and exfoliated glauconite **(C)**.

The X-ray diffraction (XRD) patterns for both the original glauconite and the synthetic versions were used to assess the modifications in the structure along with identifying the main crystalline forms. The pattern of untreated glauconite (Figure 1A) indicates the existence of galuconite as its main constituent, along with impurities like quartz, feldspar, calcite, and hematite. The glauconite form that was identified is related to the 1 M poly-type, which displays highly organized crystalline frameworks belonging to the ISII structural group. It is identifiable by a high concentration of K_2_O and has moderate expansion properties (Baldermann et al., 2022). The existence of glauconite was verified by the detection of XRD peaks at specific angles of 8.67^o^, 19.72^o^, 26.70^o^, 34.78^o^, 37.17^o^, and 61.31^o^ (Figure 1A) (Baldermann et al., 2022; Pietsch et al., 2016). The glauconite content based on the petrographic and XRD analysis is about 97.86% with total impurities content of 2.14%. The pattern of glauconite modified by DMSO exhibits major changes in its main peaks, which are centered at 8.01^o^, 19.54^o^, 26.50^o^, 34.50^o^, 36.80^o^, and 61.10^o^ (Figure 1B). This validates the expected incorporation of DMSO as an organic compound within the silicate layers. The established increase in interlayer spacing, from 10.18 Å to 11.2 Å, further supported this. The evaluation of the Mth/EXGL pattern revealed similar findings. The main peaks showed notable shifts down to lower positions, particularly at 7.47^o^, 19.52^o^, 26.5^o^, 34.4^o^, and 35.57^o^ (Figure 1C). Also, the glauconite’s crystalline silicate layers expanded significantly, increasing basal spacing to 11.81 Å. This suggested a considerable structural distortion associated with the expansion and exfoliation of these layers from each other.

**FIGURE 2:**
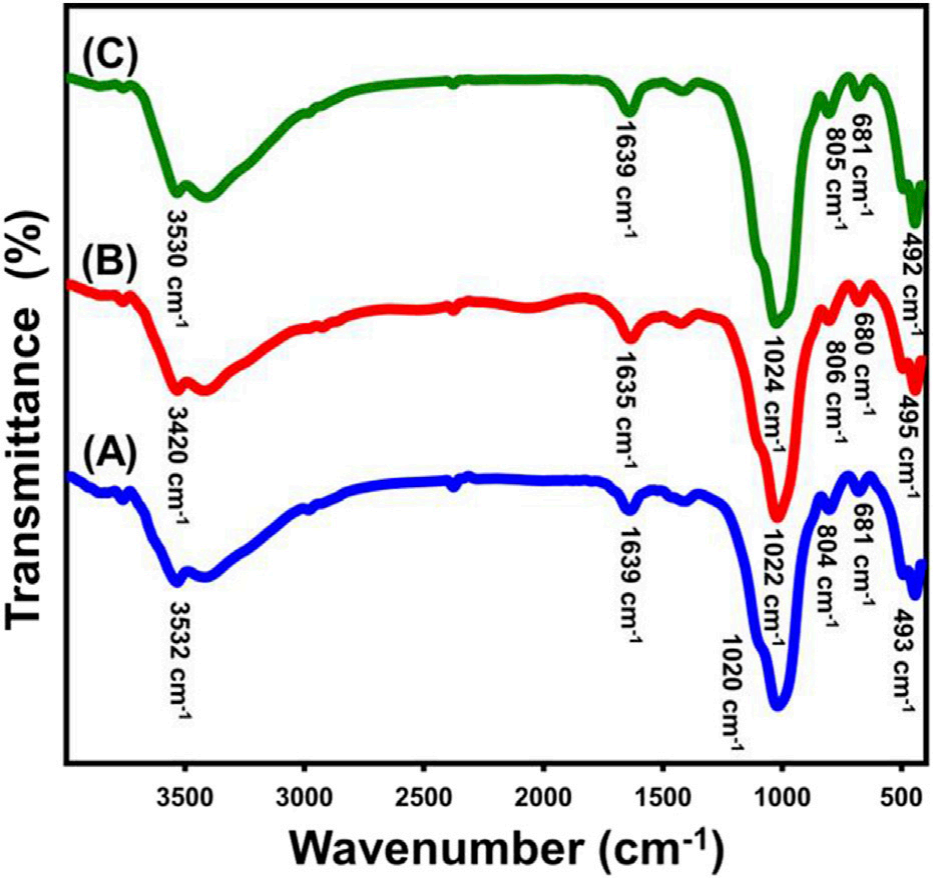
FT-IR spectra of untreated glauconite **(A)** DMSO modified glauconite **(B)**, and exfoliated glauconite **(C)**.

The changes in the basic chemical properties during the production method can be detected through examining the FT-IR spectra for both the raw glauconite along with the newly developed formed materials (Figure 2; Table. 1). The analysis of untreated glauconite revealed a spectrum revealing its aluminosilicate chemical structure (Figure 2A; Table. 1). The verification process included identifying the bands corresponding to the Si-O-Si, Si-O, Si(Al)-O-Si, Fe-OH, Si-O-Fe, and OH groups. The hydroxyl groups observed around 3,500 cm^−1^ were probably attributed to either the adsorption of water or the presence of metallic hydroxides inside the glauconite structure (Sobeih et al., 2020) (Figure 2A; Table. 1). However, the free water molecules that exist within the multilayered silicate units, particularly inside the smectite successive layers, are represented by the hydroxyl groups, which were identified around 1,600 cm^−1^ (Singla et al., 2020b) (Figure 2A; Table. 1). Moreover, the detected peaks at 800 cm^−1^ and 490 cm^-1^ indicate the existence of metallic iron at considerable content inside the structure of glauconite, which is consistent with the findings of its XRF investigation. The spectra of the samples treated with DMSO (Figure 2B; Table. 1) and Mth/EXG (Figure 2C; Table.1) displayed the same bands as the untreated glauconite, revealing no bands associated with the organic contents of these chemicals. The bands of the basic groups display some fluctuation from their positions, which might suggest the influence of embedding chemicals on the crystalline framework of glauconite. Furthermore, a distinct band of Si(Al)-O-Si exhibits a modest split at approximately 1,000 cm^−1^. This provides evidence about the distortion of the structural units (consisting of octahedron and tetrahedron subunits) caused by the partial swelling and division, also known as exfoliation, of these multilayered silicate.

**TABLE 1:** The FT-IR detectable bands of glauconite during the various lateration procedures and the corresponding chemical groups.

Absorption bands (cm^-1^)			Functional chemical groups
Glauconite	DMSO/glauconite	Metheoxy glauconite	
3,532	3,420	3,530	stretching and bending vibrations of –OH groups (Younes et al., 2021)
1,639	1,635	1,639	Interlayer water molecules (Singla et al., 2020b)
1,020	1,022	1,024	Si(Al)–O–Si asymmetric stretching (Sobeih et al., 2020; Allah et al., 2023)
804	806	805	Fe_2_^3+^ OH/Fe^2+^Fe^3+^OH bending (Sobeih et al., 2020)
681	680	681	bending vibration of Si–O and/or -OH (Sobeih et al., 2020)
493	495.6	492	Si–O–Fe^3+^ (Younes et al., 2021; El-Habaak et al., 2016)
445.6	445.02	444	Si–O–Si (Younes et al., 2021; Bruneel et al., 2021)

**FIGURE 3:**
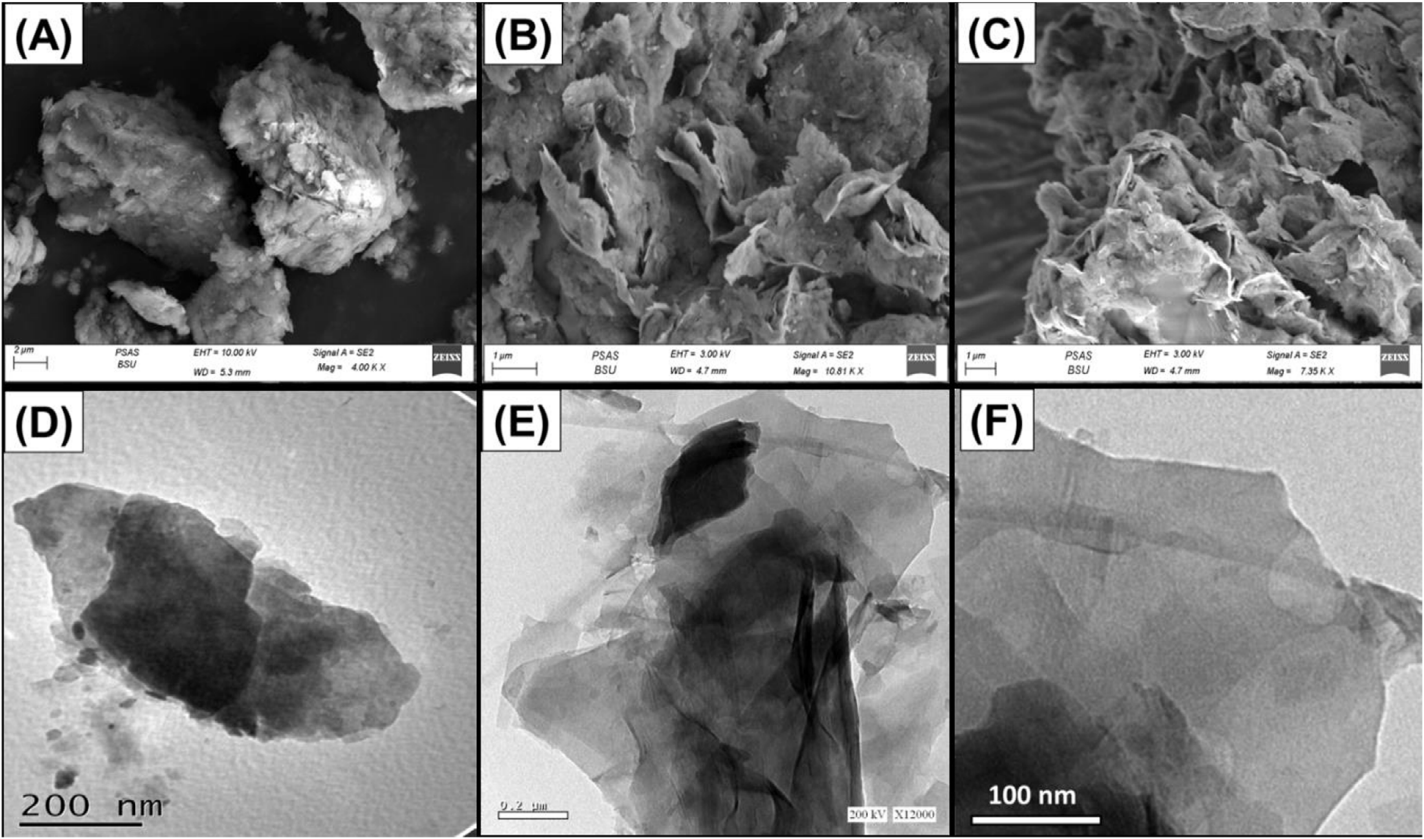
SEM raw glauconite **(A)**, SEM images of exfoliated glauconite **(B, C)**, high magnification TEM image of raw glauconite **(D)**, and high magnification TEM images of exfoliated glauconite **(E, F)**.

The changes in the structure of the material during the development processes of Mth/EXG were tracked employing the SEM and TEM images (Figure 3). The initially untreated glauconite exhibits the characteristic compacted and clustered morphology of glauconite, which is particularly prevalent as stacked and compacted strips (Figure 3A). The implantation of DMSO and then methanol within the successive silicate-layered subunits caused significant delamination along with effective separation of these layers individually (Figure 3B). After the incorporation of the surplus alcohol molecules, the exfolation behavior became substantially greater, and the glauconite particles developed separate layers and sometimes formed intersected and bended platelets resembling cornflakes (Figure 3C). The TEM images of the analyzed materials confirm the features encountered through the SEM images. The glauconite particles were noticed as large, almost oval-shaped granules that lack notable internal features (Figure 3D). After exfoliation, particles were separated into distinct layers in addition to blended particulates that exhibit cyclinederical shapes (Figures 3E, F).

### 3.2 The loading properties

#### 3.2.1 Effect of loading variables

##### 3.2.1.1 Effect of pH

**FIGURE 4:**
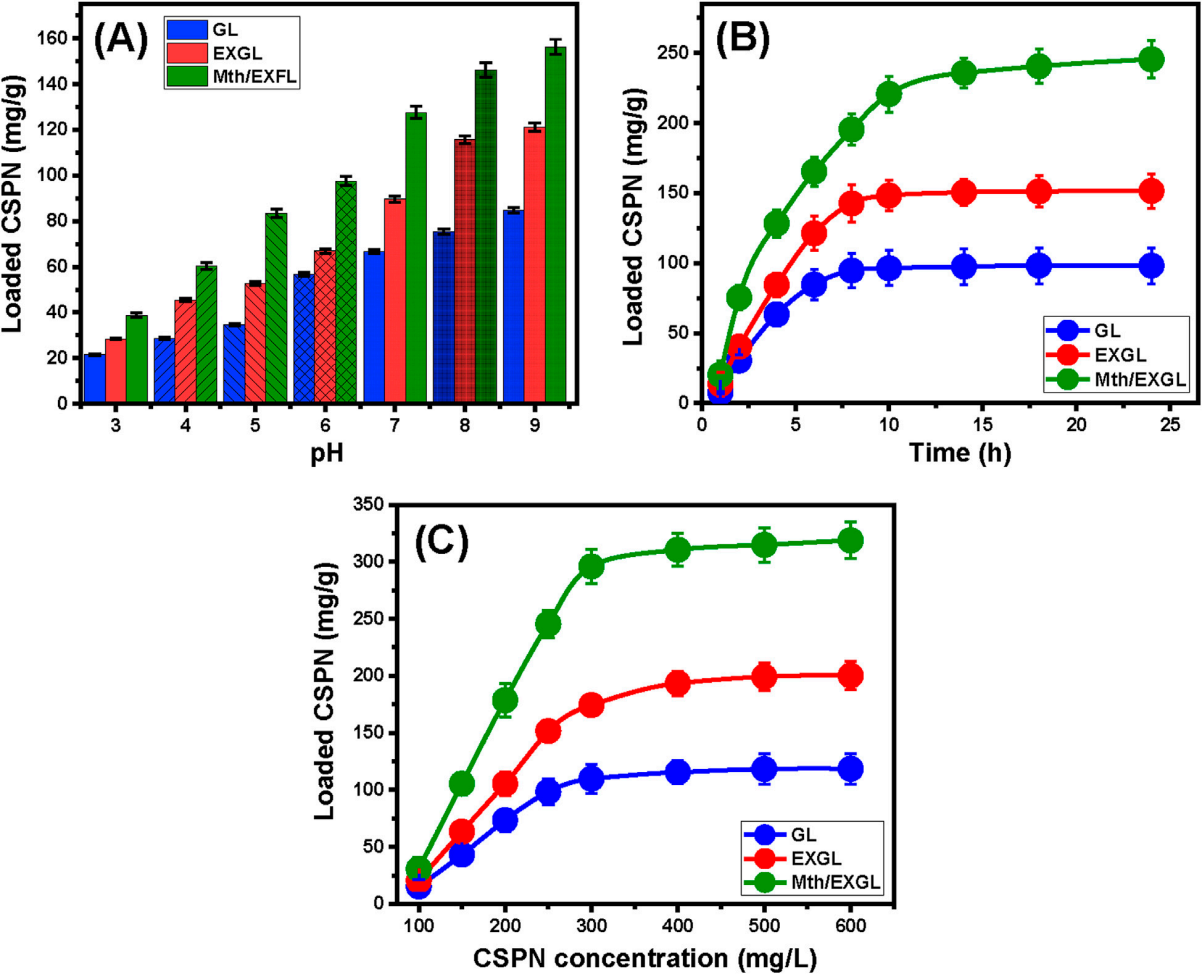
The impact of the experimental factors on the loading performances of GL, EXGL, and Mth/EXGL for CSPN including **(A)** the pH (30 mg dose, 250 mg/L CSPN content, 6 h, 20°C, and 50 mL volume), **(B)** loading duration (30 mg dose, 250 mg/L CSPN content, pH 9, 20°C, and 50 mL volume), and **(C)** CSPN starting concentration (30 mg dose, 24 h duration, pH 9, 20°C, and 50 mL volume).

The pH modifications during laboratory experimentation have a substantial influence on the electrical charges present across the exteriors of GL, EXGL, and Mth/EXGL, along with the ionization behaviors and speciation of CSPN molecules. The present investigation monitored the effect of pH on the loading efficiency of CSPN into GL, EXGL, and Mth/EXGL over the pH range between three and 8. The experiments were carried out utilizing a 30 mg dose of the solid carriers, a CSPN content of 250 mg/L, a period of 6 h, a temperature of 20°C, and an overall volume equal to 50 mL. The binding characteristics of CSPN onto GL, EXGL, and Mth/EXGL exhibited a significant rise from pH 3 (21.5 mg/g for GL, 28.4 mg/g for EXGL, and 38.8 mg/g for Mth/EXGL) to pH 9 (84.7 mg/g for GL, 121.3 mg/g for EXGL, and 156.3 mg/g for Mth/EXGL) (Figure 4A). Consequently, the loading reactions of CSPN using GL, EXGL, and Mth/EXGL are preferable in alkaline situations. The pH level greatly influences the ionizing characteristics of the CSPN molecules. The previously mentioned trend could potentially be explained by considering the distinctive speciation features of CSPN in addition to its various pKa levels (pKa = 6.56, 7.21, and 5.37). In water-based solutions, the hydrolysis and solubility of CSPN ([(NH_3_)_2_PtCl_2_]^+0^) involve the substitution of Cl ions with the existing water molecules, resulting in modified varieties with positive charges (Bruneel et al., 2021). The species that are known to have recognized varieties, cis-[Pt (OH)_2_(NH_3_)_2_] and cis-[Pt (OH) (H_2_O) (NH_3_)_2_]^+^, have pKa values of 7.21 and 5.37, respectively (Barroug and Glimcher, 2002). The varieties cis-[PtCl_2_ (H_2_O) (NH_3_)_2_]^+^ (monoaquacisplatin), cis-[PtCl (OH) (NH_3_)_2_] hydroxo complexes, and cis-[Pt (H_2_O)_2_(NH_3_)_2_]^2+^ (diaquacisplatin) have pKa values of 6.56. The recognized varieties, cis-[Pt (OH)_2_(NH_3_)_2_] and cis-[Pt (OH) (H_2_O) (NH_3_)_2_]^+^, have pKa values of 7.21 and 5.37, respectively (Barroug and Glimcher, 2002). Additionally, the pH levels affect the nature of existing charges throughout the Mth/EXGL, EXGL, and GL particulates. Under acidic conditions, the ionized form of CSPN possesses positive electrical charges and exhibits strong competitive and repellent properties with the already abundant hydronium ions on the interfaces of GL, EXGL, and Mth/EXGL. At elevated pH levels, the converse is evident; the deprotonation behaviors of the essential chemical groups across the interfaces of GL, EXGL, and Mth/EXGL led to potent electrostatic attraction for the existing CSPN-ionized species that exhibit positive charges under these conditions. As a result, it is advisable to conduct the CSPN loading processes into GL, EXGL, and Mth/EXGL at the basic pH levels.

##### 3.2.1.2 Loading duration

The loading properties of CSPN using GL, EXGL, and Mth/EXGL have been assessed at various time intervals. The duration intervals ranged from 1 h to 24 h, considering maintained levels for the effective parameters, including a dose of 30 mg, a CSPN content of 250 mg/L, a pH of 9, an ambient temperature of 20°C, and an overall volume equal to 50 mL. As the interaction duration increases progressively, the overall CSPN loading response of GL, EXGL, and Mth/EXGL shows a significant increase in either the recognized loading rates or the total quantities of loaded drug in mg/g (Figure 4B). The enhancement in loading activities could potentially be detected over a duration of 10 h for GL and EXGL and up to 14 h for Mth/EXGL. Following the aforementioned loading periods, there is negligible variation in the loading speed of the CSPN and the total amount loaded, remaining essentially steady. The steady-state of the recognized loading characteristics of the three carriers, GL (98.4 mg/g), EXGL (151.5 mg/g), and Mth/EXGL (245.5 mg/g), validated the attendance of their equilibrium states (Figure 4B). During the first loading stages, the GL, EXGL, and Mth/EXGL interfaces displayed a higher quantity of active and unoccupied sites. This resulted in a significant improvement in the speed of the loading reactions and a pronounced increase in the quantity of CSPN adsorbed (Lenz et al., 2005). As the examination period increases, CSPN is progressively occupying the available sites across the GL, EXGL, and Mth/EXGL particles. Consequently, these reactive and effective receptors get depleted, resulting in a substantial reduction in their ability to be filled or occupied with extra CSPN molecules. As a result, CSPN loading rates decreased gradually, resulting in a decline in overall efficiency for these carriers. Once all of the accessible GL, EXGL, and Mth/EXGL sites were filled with CSPN, they achieved a state of equilibrium, and their interfaces established their saturation point (Fekri et al., 2022).

##### 3.2.1.3 CSPN concentration

An investigation was conducted to examine the impact of the starting level of soluble CSPN molecules on the encapsulation properties of GL, EXGL, and Mth/EXGL. The investigation was performed under well-maintained experimental conditions, with a dose of 30 mg for the carriers, an interaction period of 24 h, a pH of 9, a temperature of 20 °C, and volumes of 50 mL. The starting contents of CSPN contribute strongly to establishing the maximum loading values of the used carriers and illustrating their equilibrium specifications. As a result, it is critical to carefully consider the loading behaviors of the suggested carriers or delivery structures being investigated. There was a considerable increase in the overall quantities of CSPN that were loaded into GL, EXGL, and Mth/EXGL whenever the trials implemented higher levels of soluble CSPN (Figure 4C). Elevated concentrations of CSPN molecules in a certain volume result in a significant increase in their mobility properties and driving forces. This eventually results in larger collisions, accompanied by chemical interactions across the binding sites of GL, EXGL, and Mth/EXGL, boosting the efficacy of the loading activities (Yadav et al., 2021). The rise in the total amount of embedded CSPN molecules, with respect to the starting content of the drug, had been recognized up to 500 mg/L (Figure 4C). The experiments conducted at CSPN concentrations above the previously specified levels yielded an ignored increment in the loaded quantities or were maintained reasonably steady. This implies that GL, EXGL, and Mth/EXGL accomplished their stabilization or saturation. The GL, EXGL, and Mth/EXGL structures successfully accomplish their highest CSPN loading capacities at 118.4 mg/g, 200.2 mg/g, and 318.7 mg/g, respectively (Figure 4C). The substantially enhanced CSPN loading properties of EXGL and Mth/EXGL in contrast with GL were assigned to a variety of parameters, such as (1) the documented rise in surface area following the modification process, (2) the organophilic characteristic of Mth/EXGL in contrast to the hydrophilicity of GL, which improves its surface affinity to the CSPN organic molecules, and (3) an appreciable rise in the numbers of the functioning loading sites after the alterations steps.

#### 3.2.2 Loading mechanism

##### 3.2.2.1 Kinetic properties

###### 3.2.2.1.1 Intra-particle diffusion properties

**FIGURE 5:**
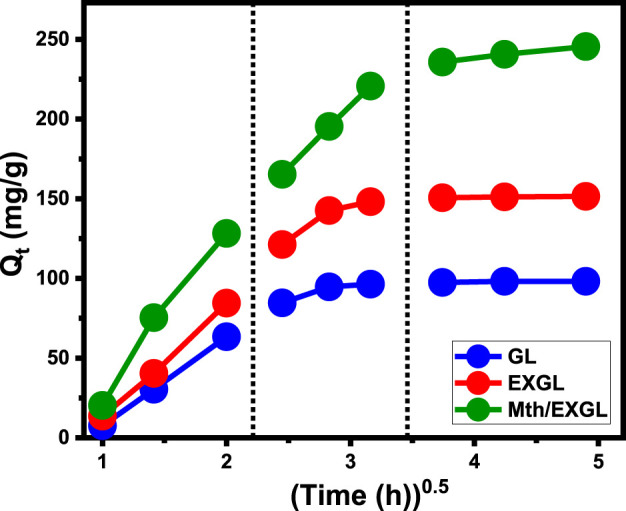
The Intra-Particle diffusion curves of the CSPN loading behaviors using GL, EXGL, and Mth/EXGL.

The inquiry examined the characteristics of intra-particle diffusion curves corresponding to the encapsulation of CSPN onto GL, EXGL, and Mth/EXGL to identify the mechanistic aspects that regulate the loading process. The obtained curves possess distinct segments and many lines that do not intersect with their starting or original spots. The analysis of CSPN loading tendencies based on the curves indicates that the retention of CSPN into GL, EXGL, and Mth/EXGL particulates is controlled by many regulatory processes instead of just relying on the intra-particle diffusion reaction (Mkilima et al., 2024; Babas et al., 2021). The studied graphs demonstrate the existence of three consecutive mechanistic steps that were effectively implemented during the loading operations. The steps involved in this process include exterior loading, intra-particle diffusion and/or layered loading, and saturating phases (Figure 5) (Altalhi et al., 2022). The first distinguishable part represents the loading processes by the external sites throughout GL, EXGL, and Mth/EXGL particles. The abundance of effective binding or receptors across the contact surfaces of GL, EXGL, and Mth/EXGL has a significant impact on this loading stage (Lin et al., 2021). The next phase pertains to the processes of intra-particle diffusion. Throughout this phase, the CSPN molecules undergo diffusion through the interior pores within the GL, EXGL, and Mth/EXGL frameworks. Subsequently, the drug molecules adhere to specific receptors located throughout the interior frameworks of these materials, without any significant role for the external loading sites (Figure 5) (Li et al., 2024). The next and third segments, designated as the saturation stage or the equilibration phases, show minimal to no change in the CSPN loading properties of GL, EXGL, and Mth/EXGL (Figure 5). The equilibrium state was ultimately accomplished by completely occupying all accessible sites and forming tightly bonded layers of CSPN across the exteriors of GL, EXGL, and Mth/EXGL via molecular associations and inter-ionic interactions (Yadav et al., 2021; Li et al., 2024).

##### 3.2.2.2 Kinetic modeling

**FIGURE 6:**
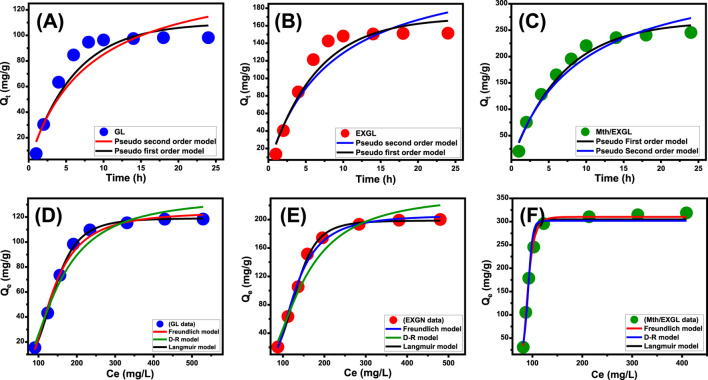
Fitting of the CSPN loading reactions with theoretical kinetic models [**(A)** GL, **(B)** EXFL, and **(C)** Mth/EXGL] and classic isotherm models [**(D)** GL, **(E)** EXGL, and **(F)** Mth/EXGL].

The loading processes of CSPN using GL, EXGL, and Mth/EXGL were discussed based on their kinetic behaviors, utilizing the parameters and assumptions of pseudo-first-order (PFO) (Equation 3) and pseudo-second-order (PSO) (Equation 4) models (Figures 6A–C; Table 2). The nonlinear fitness of the experimental findings with the two models, employing both the corresponding correlation coefficient (*R* ^*2*^) and Chi-square (*X* ^*2*^) results, determined the alignment and agreement levels (Fekri et al., 2022).

$$Q_{t\;}=Q_{e}\;\left(1-e^{{-k}_{1}.t}\right)$$ (3)

$$Q_{t}=\frac{Q_{e\;}^{2}k_{2}t}{1+Q_{e}k_{2}t}$$ (4)

**TABLE 2:** The estimated mathematical parameters of loading kinetic, classic isotherm, advanced isotherm, and release kinetic models.

Model	Parameters	GL	EXGL	Mth/EXGL
Kinetic models				
Pseudo-first-order	K_1_ (min^-1^)	0.166	0.157	0.146
	Qe _(Cal)_ (mg/g)	109.77	169.4	267.2
	R^2^	0.94	0.95	0.97
	X^2^	2.79	2.69	2.49
Pseudo-second-order	k_2_ (g mg^-1^ min^-1^)	8.3 × 10^−4^	5.11 × 10^−4^	3.01 × 10^−4^
	Qe _(Cal)_ (mg/g)	152.3	235.8	373.8
	R^2^	0.91	0.94	0.96
	X^2^	3.8	3.96	3.69
Isotherm models				
Langmuir	Qmax (mg/g)	119.1	206.7	324.6
	b(L/mg)	1.94 × 10^−8^	6.19 × 10^−9^	1.5 × 10^−10^
	R^2^	0.99	0.99	0.98
	X^2^	0.025	0.14	1.64
	RL	0.99	0.99	0.99
Freundlich	1/n	0.34	0.30	0.12
	k_F_ (mg/g)	124.1	206.3	310.2
	R^2^	0.98	0.98	0.99
	X^2^	0.84	1.01	0.36
D-R model	β (mol^2^/KJ^2^)	0.0062	0.0066	0.0088
	Q_m_ (mg/g)	136.1	236.6	330.7
	R^2^	0.98	0.97	0.98
	X^2^	0.74	2.27	2.51
	E (KJ/mol)	8.95	8.70	7.53
Monolayer model of one energy	n	4.53	5.27	7.2
	Nm (mg/g)	26.3	38.4	45.5
	Q_(sat)_ (mg/g)	119.3	202.4	327.7
	∆E (kJ/mol)	−10.06	−10.2	−6.8

	EXGL		Mth/EXGL	
	Acetate buffer (pH 5.5)	Phosphate buffer (pH 7.4)	Acetate buffer (pH 5.5)	Phosphate buffer (pH 7.4)
Release kinetics				
Zero-order	0.69	0.67	0.66	0.57
First order	0.91	0.92	0.98	0.98
Higuchi	0.89	0.86	0.87	0.80
Hixson-Crowell	0.85	0.88	0.93	0.94
Korsmeyer-peppas	0.84	0.84	0.82	0.83
n	0.63	0.51	0.49	0.57

It is feasible to confirm that the loading processes of CSPN utilizing GL (Figure 6A), EXGL (Figure 6B), and Mth/EXGL (Figure 6C) follow the kinetic features and principles of the PFO theory according to the estimated values of both *R* ^*2*^ and *X* ^*2*^. The agreement between the analytical equilibrium loading results (GL (98.2 mg/g), EXGL (151.5 mg/g), and Mth/EXGL (245.5 mg/g)) and the theoretically computed values as the model’s fitting parameter (Qe = 109.7 mg/g (GN), 169.4 mg/g (EXGL), and 267.2 mg/g (Mth/EXGL)) confirms the described kinetic findings and the fitting results (Table. 2). The kinetic basics of this model reflect the physical loading processes of CSPN, which include van der Waals forces and/or electrostatic attraction (Sherlala et al., 2019; Huang et al., 2018). Despite this, the CSPN loading behavior and PFO hypotheses agree more closely than with PSO principles. The results still show a satisfactory level of fit with the PSO model. As a result, additional chemisorption processes, such as electron exchanges, chemical complexes, and hydrogen bonds, could have a supportive effect throughout the CSPN loading process (Mkilima et al., 2024; Sherlala et al., 2019). The development of a chemically bonded layer of CSPN might facilitate the complex interactions between chemical and physical processes. This layer could potentially serve as a starting point for developing further layers of loaded CSPN through physical processes (Jasper et al., 2020; Huang et al., 2014).

##### 3.2.2.3 Equilibrium properties

###### 3.2.2.3.1 Classic isotherm modeling

The equilibrium aspects of the bonding mechanisms of CSPN onto GL, EXGL, and Mth/EXGL have been examined using three of the frequently employed isotherm approaches: Langmuir (Equation 5), Freundlich (Equation 6), and Dubinin-Radushkevich (D-R) (Equation 7). The *R* ^*2*^ and *X* ^*2*^ levels were determined by nonlinear regression analysis to assess the extent of agreement with these models (Table 1).

$$Q_{e}=\frac{Q_{\max}\;bC_{e}}{\left(1+bC_{e}\right)}$$ (5)

$$Q_{e}=K_{f}C_{e}^{1/n}$$ (6)

$$Q_{e}=Q_{m}e^{-\beta {ɛ}^{2}}$$ (7)

The statistically relevant values of *R* ^*2*^ and *X* ^*2*^ suggest that the Langmuir equilibrium theory shows a better representation of the loading behaviors of CSPN into GL (Figure 6D) and EXGL (Figure 6E) than the Freundlich concept. This suggests that CSPN molecules were loaded and bonded to the interfaces of GL and EXGL in a uniform manner controlled by the homogenously distributed receptors, forming a monolayer (Dinu et al., 2017). However, the loading of CSPN into Mth/EXGL (Figure 6F) follow the equilibrium behavior of Freundlich isotherm suggesting heterogeneous and multilayered loading of the drug molecules across the surface of methoxy modified version of exfoliated glauocnite. Furthermore, the Langmuir simulation indicates that the numerical values of the equilibrium factor (RL) were below one, indicating that CSPN exhibits favorable encapsulation properties into GL, EXGL, and Mth/EXGL particles (Table. 2). Based on the evaluation of Langmuir’s equilibrium, the expected maximum CSPN loading qualities (Q_max_) of GL, EXGL, and Mth/EXGL were 119.1 mg/g, 206.7 mg/g, and 324.6 mg/g, respectively (Table. 2).

The D-R model precisely depicts the fluctuations in energy distribution across the interfaces of GL, EXGL, and Mth/EXGL during CSPN loading processes, independent of their homogeneity or heterogeneity (Dinu et al., 2017). The adsorption energy (E) obtained from the D-R modeling offers valuable insights into the underlying mechanistic processes that regulate the loading of CSPN, irrespective of their physical or chemical characteristics (Dinu et al., 2017; Dhaouadi et al., 2021). Whenever the energy levels are below 8 kJ/mol, there are significant physical reactions occurring. If the energy levels fall between 8 and 16 kJ/mol, weak chemical or complicated chemical and physical reactions dominate. Higher levels, exceeding 16 kJ/mol, indicate the presence of strong chemical reactions. The calculated values of E for the CSPN loading activities employing GL, EXGL, and Mth/EXGL were 8.95 kJ/mol, 8.7 kJ/mol, and 7.5 kJ/mol, respectively (Table 2). The E results suggested the existence of complex physical and chemical pathways throughout the CSPN loading reactions into GL and EXGL. However the modification with methanol induced the physical process to be of the dominant effect during the loading of CSPN into Mth/EXGL particles.

###### 3.2.2.3.2 Advanced equilibrium studies

**FIGURE 7:**
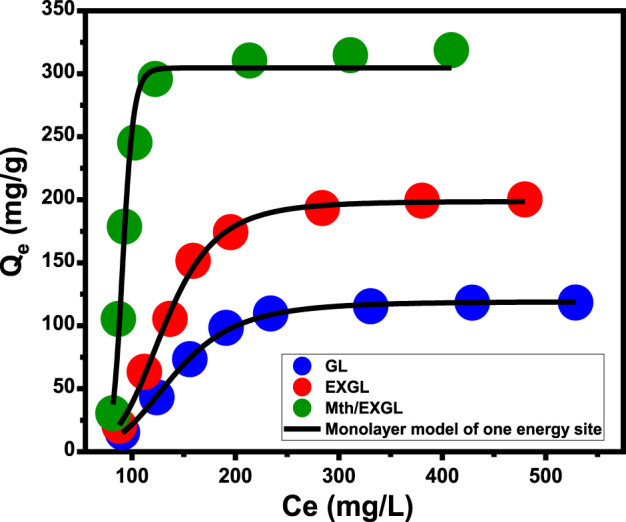
Fitting of the CSPN loading reactions with advanced Monolayer equilibrium model of one energy site.

The study investigated the application of advanced isotherm hypotheses based on statistical physics concepts to determine the mechanistic process that affected the loading of CSPN into GL, EXGL, and Mth/EXGL particulates. The characteristics of the CSPN loading reactions were represented and explained by the monolayer model with a single site of energy (Equation 8) (Figure 7; Table 2). The previously described model possessed the best *R* ^*2*^ value along with the lowest root mean square error (RMSE).

$$Q=nN_{o}=\frac{nN_{M}}{1+{\left(\frac{C1/2}{Ce}\right)}^{n}}=\frac{Q_{o}}{1+{\left(\frac{C1/2}{Ce}\right)}^{n}}$$ (8)

In order to further illustrate the loading reactions and regulated mechanisms, the theoretical parameters that were derived from the model have been estimated and assessed. The steric characteristics, such as the densities of the reactive and effective receptors (Nm _(CSPN)_), estimated numbers of loaded CSPN per only one receptor (n _(CSPN)_), and the loading performances of the carriers after their saturation with the drug molecules (Q_sat_), are presented in Table 1 together with the loading energy (E). The densities of the binding receptors (Nm _(CSPN)_) significantly increased following the exfoliation and scrolling transformations. The estimated values showed an upward trend, rising from 26.3 mg/g for GL to 38.4 mg/g for EXGL, and then to 45.5 mg/g for Mth/EXGL (Table. 2). The results confirm a significant increase in the overall quantity of effective loading sites following the exfoliation with improved dispersion characteristics and surface area. The key factors underlying these behaviors involve (1) the rise in the surface area, the elevation in the exposed reactive chemical groups, specifically the siloxane groups, (2) enhancement in the reactivity of the silicate interfaces that result from the transformation of GL into semi-crystalline or amorphous structures, and (3) the integration of extra-active functional groups corresponding to hybridization with methanol. These transformations promote the formation of highly interactive interfaces between the exteriors of the GL-derived carriers and the tested soluble CSPN molecules. Therefore, loading properties of the Mth/EXGL framework at its saturation condition (Q_sat_ = 327.7 mg/g) were found to be significantly better than those of EXGL (Q_sat_ = 202.4 mg/g) and GN (Q_sat_ = 119.3 mg/g) (Table. 2). Additionally, the methanol modification process improved the loading performance of each reacting receptor or binding site. Specifically, each binding site throughout the Mth/EXGL structure can accommodate up to 8 CSPN molecules, but EXGL and GN can retain 6 and 4 molecules, respectively. The determination of the n _(CSPN)_ value at a level above one indicates the existence of multi-molecular mechanisms throughout the trapping of CSPN into GL, EXGL, and Mth/EXGL. Moreover, it signifies the vertical orientations of the loaded CSPN molecules (Sellaoui et al., 2020; Dhaouadi et al., 2020).

Using Equation 9, the loading energies (E) were determined and applied in estimating the types of mechanisms, either physical or chemical, that affect the trapping of CSPN into GL, EXGL, and Mth/EXGL.

$$∆E=-RT\;\ln\left(\frac{S}{C1/2}\right)$$ (9)

**FIGURE 8:**
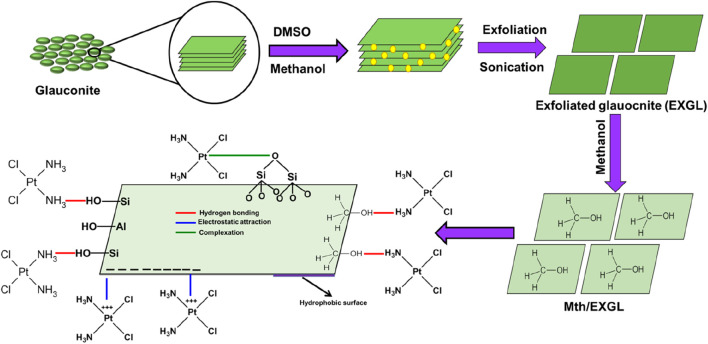
Schematic diagram for the CSPN loading mechanism into the glauconitic sheets and methanol hybridized sheets.

The CSPN loading reaction demonstrated binding energies during their encapsulations into GL, EXGL, and Mth/EXGL equal to −10.06 kJ/mol, −10.2 kJ/mol, and −6.8 kJ/mol, respectively (Table 2). The prior findings suggest that the incorporation of CSPN into GL, EXGL, and Mth/EXGL could be achieved by means of physical processes involving dipole bond forces (with an energy range of 2–29 kJ/mol), slightly van der Waals forces (with an energy range of 4–10 kJ/mol), electrostatic attraction (with an energy range of 2–50 kJ/mol), and hydrogen bonds (with an energy below 30 kJ/mol) (Sellaoui et al., 2020; Dhaouadi et al., 2020). The loading process and mechanisms were represented schematically in Figure 8.

### 3.3 *In vitro* release profiles

**FIGURE 9:**
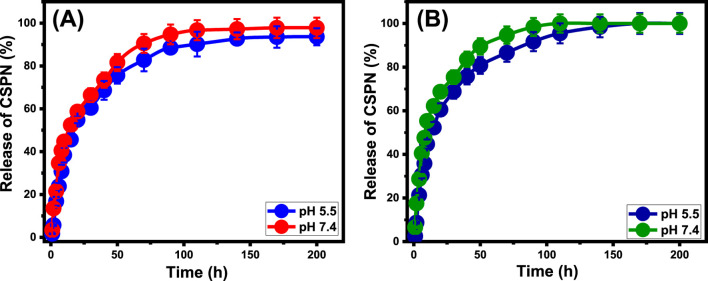
The releasing profiles of CSPN out of the structures of EXGL **(A)** and Mth/EXGL **(B)** at pH 5.5 and pH 7.4.

The CSPN-releasing processes of EXGL and Mth/EXGL have been assessed using specified quantities of CSPN molecules released through two specific buffers: phosphate (pH 7.4) and acetate (pH 5.5). These buffers have been selected to mimic the conditions associated with malignant cells (Figure 9). The loaded CSPN molecules were diffused at adequate levels out of EXGL and Mth/EXGL, implementing both of the buffered solutions that had been tested. The loading processes’ marked rates varied significantly, with noticeable increases in the release period. EXGL as well as Mth/EXGL particulates, demonstrate remarkable levels of sustained drug release throughout the early release phases (Figure 9). This observation is supported by notable variations in the amount of drug expelled. After specific periods, the tracked CSPN release rates significantly decreased. Furthermore, there were no noticeable fluctuations in the release rates, indicating a condition of equilibrium throughout the last period of the investigation.

The quick desorption and liberation of CSPN from both loosely bonded and physically trapped molecules, through the exterior active loading receptors of EXGL and Mth/EXGL, likely causes the rapid migration of CSPN during the initial phases of release (AbouAitah et al., 2021; Wang et al., 2021; Amini et al., 2021). After the surface-loaded and weakly entrapped CSPN molecules were fully released from the EXGL and Mth/EXGL structures, different types of complexes and strongly chemically bound CSPN molecules had a strong negative effect on the diffusion activities. Additionally, the CSPN molecules, trapped within the internal pores of the Mth/EXGL’s framework, adversely affected the described diffusion rates (Abukhadra et al., 2020; Elboraey et al., 2021). Additionally, the release processes of CSPN from EXGL and Mth/EXGL were much higher in the basic environment (pH 7.4) compared to those under more acidic conditions (pH 5.5).

The discharge of CSPN from EXGL across phosphate as well as acetate solutions has been observed over approximately 200 h; however, the precise amounts of complete release were not ascertained (Figure 9A). After a duration of 200 h, the highest quantities of CSPN discharged by EXGL at pH 5.5 and pH 7.4 are 93.6% and 97.8%, respectively (Figure 9A). The releasing characteristics of the Mth/EXGL structure demonstrate higher rates compared to the EXGL structure. The Mth/EXGL released almost 50% of the originally loaded CSPN after prolonged exposure to a pH of 5.5 for approximately 15 h and a pH of 7.4 for approximately 8 h (Figure 9B). The complete release was detected after 110 h at pH 7.4 and after 170 h at pH 5.5 (Figure 9B). Several factors contribute to the observed rise in the releasing rate following the exfoliation of GL into EXGL and the methanol integration phase. Firstly, the exfoliation technique reduces the amount of entrapped CSPN ions inside the interlayer space of unprocessed GL. Secondly; exfoliation activates and enhances the exposure of the reactive chemical groups, which in turn trigger the surficial and physical loading of the drug ions. Third, using methanol in the EXGL hybridization process reduces the number of hydrogen bonds directly formed between the aluminosilicate framework and CSPN functional groups. Lastly, the incorporated methanol molecules supply the exterior of EXEGN with significant quantities of negative hydroxyl groups that operate as inactive centers for weak electrostatic interactions with the drug ions (Lee et al., 2019; Kaidar-Person et al., 2018). Furthermore, the significant quantity of loaded CSPN per single active site (n _(CSPN)_ = 5.27) across the exterior of Mth/EXGL indicates that the drug molecules have a higher tendency to aggregate on the Mth/EXGL in comparison with EXGL (n _(CSPN)_ = 7.2).

This aggregation leads to an increased release rate for the drug that has been loaded. In numerous cases, the suggested courses for malignancy treatment recommend administering CSPN molecules immediately through the patients’ organs as a form of chemotherapy at a prolonged and gentle rate. This approach facilitates prolonged contact and interactions between the administered medication and the malignant cells (Alqahtani et al., 2023b). Under specified conditions, it is advisable to provide the prescribed therapeutic dose of the drug at regular intervals using rapid delivery methods with a sudden and brief duration. Studies have demonstrated the effectiveness of EXGL and Mth/EXGL delivery structures in loading and releasing CSPN compounds in controlled amounts.

### 3.4 *In vitro* release kinetics

Kinetic evaluations were conducted to analyze the release characteristics of CSPN through the developed EXGL and Mth/EXGL structures, serving as indicators of regulated mechanistic processes. The release pathways have been modeled using zero-order (Z-O) (Equation 10), first-order (F-O) (Equation 11), Higuchi (H-G) (Equation 12), Hixson-Crowell (H-C) (Equation 13), and Korsmeyer-Peppas (K-P) (Equation 14) kinetics. The mathematical modeling methods were evaluated based on the fitting extent of the linear regression analysis (Tian et al., 2020).

$$W_{t}-W_{0}=K_{0}.t$$ (10)

$$\ln\left(\;W_{\infty }/W_{t}\right)=K_{1}.t$$ (11)

$$W_{t}=K_{h}t^{1/2}$$ (12)

$$W_{o}^{1/3}-W_{t}^{1/3}=K_{HC}t$$ (13)

$$W_{t}/\;W_{\infty }=K_{p\;}$$ (14)

The zero-order kinetic theory suggests the diffusion of CSPN through EXGL as well as Mth/EXGL at consistent speeds, irrespective of the quantity of CSPN preloaded (Othman et al., 2021). The effectiveness of releasing is significantly influenced by the dosages of CSPN, according to the F-O theory. The Higuchi kinetics theory suggests that diffusion processes play a vital role in releasing behaviors (Nandi et al., 2020; El-Hamshary et al., 2019). The diffusion systems, which exhibited Higuchi kinetics, proceeded at a consistent speed that was lower than the original dose of CSPN. Furthermore, the materials utilized must have the ability to sink, and neither their swelling nor solubility degrees influence the releasing mechanism (Othman et al., 2021). The kinetic principle of the Hixson-Crowell hypothesis depends on erosion trails, and the surface area and particle dimensions of the carriers significantly influence their releasing characteristics (Othman et al., 2021). The Korsmeyer-Peppas theory suggests that release mechanisms use a combination of diffusion and erosion processes that function in parallel.

**FIGURE 10:**
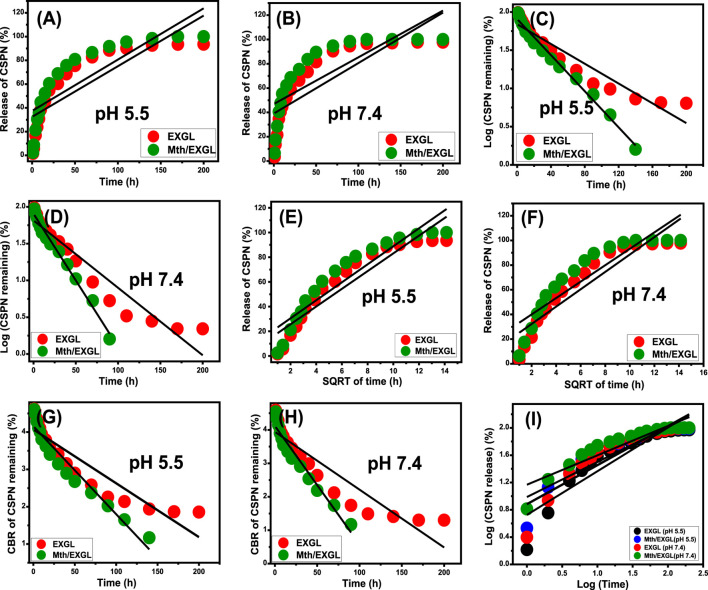
Fitting of the CSPN releasing results from EXGL and Mth/EXGL with zero-order **(A, B)**, First-order **(C, D)**, Higuchi **(E, F)**, Hixson-Crowell **(G, H)**, and Korsmeyer-Peppas **(I)** models.

The determined fitting levels (depending on the established determination coefficients) with respect to the buffered phosphate and acetate fluids indicate that the kinetic aspects of the F-O model (Figures 10C, D; Table.2) are better at illustrating the CSPN-releasing tendencies through EXGL and Mth/EXGL than the Z-O theory (Figures 10A, B; Table.1). This suggested that the overall amount of preloaded CSPN medicine significantly influences the releasing properties that encompass both EXGL and Mth/EXGL. Also, the results of releasing experiments of EXGL and Mth/EXGL display noticeable matches with the hypotheses of Higuchi (Figures 10E, F; Table.1) and Hixson-Crowell (Figures 10G, H; Table.1) models. Consequently, the releasing tendencies of EXGL and Mth/EXGL as CSPN delivery structures included a mix of diffusion and erosion processes. The eroded characteristic might be ascribed to the partial breakdown of clay minerals under elevated pH values. The strong correlation between the releasing activities of CSPN and the kinetic theories suggested by the Korsmeyer-Peppas model indicates that diffusion reactions are the main process, alongside the eroded processes, especially inside the phosphate fluids (Figure 10I; Table. 1). The calculated values of the diffusion exponent (n) of EXGL (0.63 (acetate) and 0.51 (phosphate)) and Mth/EXGL (0.49 (acetate) and 0.57 (phosphate)) verified non-Fickian transport activities, which match previously recognized kinetic findings about the combined effects of diffusion alongside erosion processes (Swain et al., 2024a).

### 3.5 Cytotoxicity properties

**FIGURE 11:**
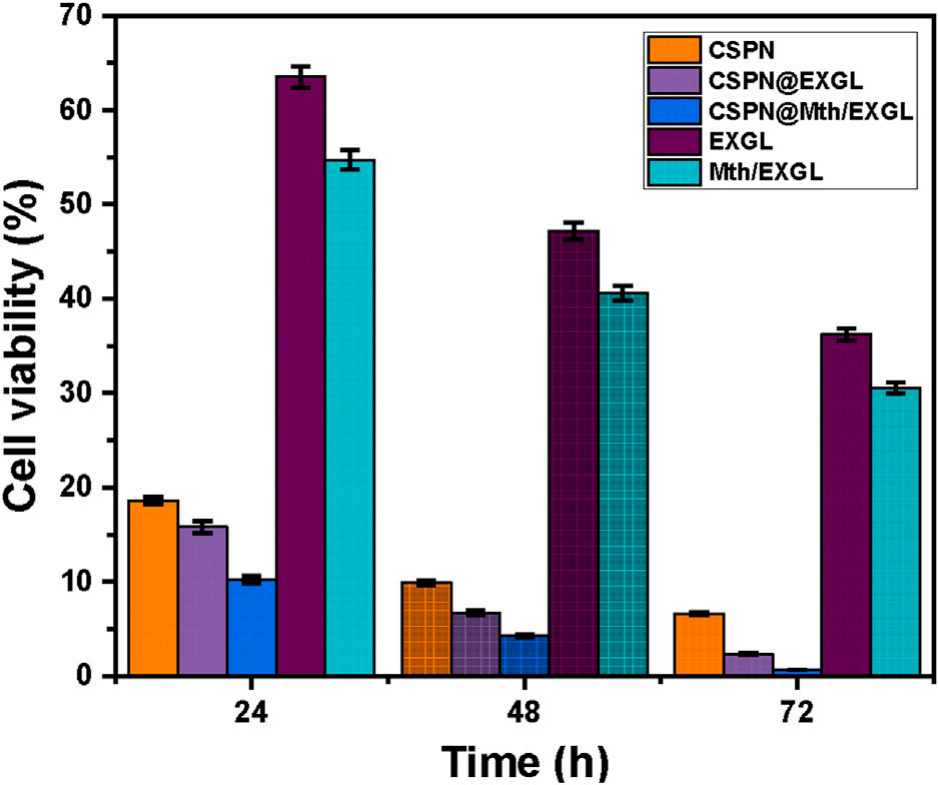
Cytotoxicity properties of free CSPN, EXGL, Mth/EXGL, and CSPN loaded EXGL, and CSPN loaded Mth/EXGL on HeLa cells.

The efficacy of liberated CSPN in suppressing the proliferation of tumors was assessed in comparison to that of free EXGL and Mth/EXGL particles, in addition to their CSPN-loaded versions, during the treatment of human cervical epithelial malignancies (HeLa) (Figure 11). The CSPN drug demonstrated severe cytotoxicity towards HeLa carcinoma cells, especially when delivered at the highest dosage of 1 μg/mL. The influence of prolonged incubation durations on cell viability increased gradually, leading to a remarkable decline in cell viability levels to 18.6% after 24 h, 9.9% after 48 h, and 6.6% after 72 h (Figure 11). Encapsulating the CSPN medicine within both EXGL and Mth/EXGL significantly enhanced its efficacy in treating cancer. The CSPN-loaded EXGL demonstrated an important improvement in its inhibition activities against the cancerous cells. More precisely, the cytotoxic impact increased by 15.8%, 6.7%, and 2.3% during incubation periods of 24 h, 48 h, and 72 h, respectively (Figure 11). Furthermore, the use of Mth/EXGL as a transporter for CSPN resulted in a significant improvement in its therapeutic efficacy for the studied HeLa cells. Administering 1 μg/mL of CSPN-loaded Mth/EXGL resulted in cell viability levels of 10.2%, 4.3%, and 0.65% after 24 h, 48 h, and 72 h, respectively. The unbound EXGL and Mth/EXGL have also shown significant anticancer activity towards HeLa carcinoma cells. The cell vitality was quantified after treating the cells with 1 μg/mL of EXGL and Mth/EXGL for 72 h, resulting in values equal to 36.2% and 30.5%, respectively. The recognized cytotoxic properties can be attributed to the considerable reactivity of the exfoliated glauconite nano-layers and their advanced form as nanorods, along with the verified oxidative properties of clay nanomaterials resulting from the existence of metallic impurities, particularly iron, in the glauconite framework. Furthermore, the findings demonstrated that the one-dimensional framework of GN has a significant influence on its cellular toxicity as a possible therapy for carcinoma. As a result, the findings regarding loading, release, and cytotoxic properties reinforce the effective application of EXGL and Mth/EXGL as CSPN drivers throughout the chemotherapy of carcinogenic HeLa cells.

## 4 Conclusions

The exfoliated galuconite mono-silicate sheets were effectively functionalized by methanol (Mth/EXG) as an effective carrier of cisplatin with enhanced surface and biological activities. The Mth/EXG carrier demonstrated enhanced loading capacities (327.7 mg/g) as compared to EXGL (202.4 mg/g) and raw glauocnite (119.3 mg/g) (30 mg dose, 24 h duration, pH 9, 20 °C, 600 mg/L CPN concentration, and 50 mL volume). This was attributed to the considerable changes in the surface chemistry, organophilic properties, and density of loading sites (45.5 mg/g (Mth/EXGL), 38.4 mg/g (EXGL), and 26.3 mg/g (GL)). The energetic investigations (<8 kJ/mol) suggested physical loading of CSPN into Mth/E.G., Also, the release behavior reflects continuous and regulated properties up to 170 h, considering the complex impact of erosion and diffusion mechanisms. Mth/EXGL exhibits considerable anticancer activity against HeLa cells (30.5% cell viability) and also enhances the cytotoxic impact of the loaded drug from 6.6% to 0.65% (cell viability). Therefore, the structure is highly recommended to be applied as a delivery system for CSPN chemotherapy and anticancer agents, which can be supported by further deep *in vivo* biological studies.

## Data Availability

The datasets presented in this study can be found in online repositories. The names of the repository/repositories and accession number(s) can be found in the article/supplementary material.
